# Influencing Factors on the Healing Performance of Microcapsule Self-Healing Concrete

**DOI:** 10.3390/ma14154139

**Published:** 2021-07-25

**Authors:** Yanju Wang, Zhiyang Lin, Can Tang, Wenfeng Hao

**Affiliations:** 1 Beijing Institute of Aeronautical Materials, Aviation Engine Corporation of China, Beijing 100095, China; yjwangbiam@163.com; 2 Jiangsu Surveying and Design Institute of Water Resources Co., Ltd., Yangzhou 225127, China; zylin2021vip@163.com; 3 Faculty of Civil Engineering and Mechanics, Institute of Structural Health Management, Jiangsu University, Zhenjiang 212013, China

**Keywords:** self-healing concrete, microcapsule, influencing factors, compressive strength, sodium silicate, healing rate

## Abstract

The amounts of the components in a microcapsule self-healing system significantly impact the basic performance and self-healing performance of concrete. In this paper, an orthogonal experimental design is used to investigate the healing performance of microcapsule self-healing concrete under different pre-damage loads. The strength recovery performance and sound speed recovery performance under extensive damage are analyzed. The optimum factor combination of the microcapsule self-healing concrete is obtained. Scanning electron microscopy (SEM) and energy dispersive spectroscopy (EDS) are carried out on the concrete samples before and after healing to determine the healing mechanism. The results show that the healing effect of self-healing concrete decreases with an increase in the pre-damage load, and the sound speed recovery rate increases with an increase in the damage degree. The influence of the sodium silicate content on the compressive strength and compressive strength recovery rate of the self-healing concrete increases, followed by a decrease. The optimum combination of factors of the microcapsule self-healing system is 3% microcapsules, 30% sodium silicate, and 15% sodium fluosilicate. The results can be used for the design and preparation of self-healing concrete.

## 1. Introduction

Self-healing concrete is used to prevent concrete deterioration and improve durability [1,2,3]. Since there are insufficient human and material resources for damage detection and the timely repair of concrete components, scholars proposed a self-healing concrete with bionic characteristics. Microcapsule self-healing composites, a research hot spot in the field of self-healing, have received increasing attention for use in cement-based materials [4,5,6,7].

Since White et al. [8] first developed concrete microcapsules with a self-healing ability using injection techniques and polymerization, scholars have conducted numerous studies on microcapsule self-healing concrete using different methods [9,10,11,12,13]. In recent years, researchers focused on the performance and technology of microencapsulated self-healing concrete. Jose [14] introduced calcium nitrate as a self-healing material to improve the microencapsulation process and the mechanical properties of the microencapsulated concrete. The mix proportion of the concrete was determined by experiments, and the self-healing efficiency of the self-healing concrete was evaluated. Perez [15] innovatively used nano-silica and amine-based materials with good cement compatibility as wall and core materials of microcapsules, respectively, to form a relatively stable concrete self-healing system. Wang et al. [16] used bentonite and an expansive agent as core materials and swelling resin as a wall material. The permeability was evaluated to determine the optimal process and the optimal components of the microcapsules. In addition, the effects of the crack width and microcapsule dosage on the repair performance were investigated. Zhu et al. [17] used basalt fiber/cement as the self-healing material and determined the optimum mix proportion of the composite material using the ultrasonic time, amplitude, and frequency as indices. Giannaros et al. [18] studied the repair performance of self-healing mortar specimens with different contents of the microcapsules and curing agent. Zhang et al. [19] used the mercury intrusion and nitrogen adsorption methods combined with micro-computed tomography (μ-CT) to characterize the pore structure of the microencapsulated cement-based materials containing epoxy resin. It was found that the pore structure was significantly improved after healing. The above studies demonstrated the effectiveness of the performance recovery of self-healing concrete with microcapsules and focused on the appropriate dosage of the microcapsules, laying a foundation for subsequent large-scale experimental applications.

Other studies focused on the diversity of healing conditions and mechanisms of self-healing concrete with microcapsules. Sun et al. [20] prepared melamine urea formaldehyde (MUF) microcapsules by in situ polymerization and studied the self-healing performance of asphalt concrete pavement. Kosarli et al. [21] synthesized cement-based self-healing microcapsules with epoxy resin as the core material, analyzed the self-healing process of the microcapsules in the concrete using the capillary dynamics principle, and established a new self-healing evaluation method using the seepage structure parameters, pore structure parameters, and adsorption–desorption curve as indices. Lv et al. [22] focused on the healing performance of self-healing concrete with epoxy resin microcapsules under a chloride and strong acid attack. Wang et al. [23] combined microcapsule self-healing and microbial self-healing. The microcapsules protected the bacteria producing calcium carbonate, thus filling the cracks. Leeys et al. [24] used polyvinyl alcohol (PVA) fibers and precipitation crystallization for self-healing. The PVA self-healing fibers were wrapped around the precipitation crystallization material, resulting in a good self-healing effect. Hong et al. [25] established a new test system for self-healing concrete to evaluate damage and cracking. X-ray μ-CT was used to analyze the healing mechanism of two types of microcapsule self-healing systems. Both systems provided a good crack healing performance in concrete. The reviewed studies combined different principles to explore the healing mechanism of microencapsulated self-healing concrete in different environments. Thus, different working conditions and various evaluation indicators should be used to evaluate the self-healing performance comprehensively and objectively.

However, the amounts of the components in the microcapsule self-healing system and the components’ influences on the basic properties and self-healing properties of concrete remain unclear. In this paper, a microcapsule system is used in cement-based composites, and an orthogonal test is used to analyze the healing performance for different pre-damage loads. The strength recovery performance and sound speed recovery performance under extensive damage are analyzed using an orthogonal test. The optimum factor combination of the microcapsule self-healing cementitious composite system is obtained. Scanning electron microscopy (SEM) and energy dispersive spectroscopy (EDS) are conducted on the concrete samples before and after healing to determine the healing mechanism of the microcapsule self-healing concrete.

## 2. Specimen Preparation

A Wj-3 extrusion spheronizer (Panfeng Drying Equipment Co., Ltd, Changzhou, China) was used to prepare the microcapsules. The main steps included preparation of the wet material, extruding the strips, rolling the balls, spray coating, and hot-air drying. The first three steps focused on forming the pellets, and spray coating was used to combine the core and the wall. An appropriate coating solution was used. Due to the relatively high viscosity of the ethyl cellulose solution used in the capsule wall, the dissolving agent has volatile components. Thus, that material is prone to thermosetting, and hot-air drying was needed to prevent mutual adhesion. The preparation of the wet material was the first step and determined the composition of the repair material in the core material. Portland cement and sodium silicate, which react with calcium hydroxide, were added to the self-healing microcapsules, representing the core materials. This composition facilitates the comparison with subsequent experiments.

**Figure 1:**
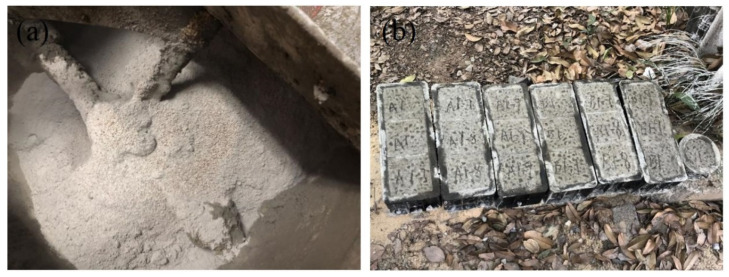
Preparation of the microencapsulated self-healing concrete cube specimens. (**a**) Adding the microcapsules. (**b**) Cube test piece before demolding.

Concrete specimens (100 mm × 100 mm × 100 mm cubes) were prepared. The fixed water binder ratio was 0.54, the fineness modulus of the sand was 3.0–2.3, and the particle size of the gravel was about 3–5 mm. The cement properties are listed in Table 1 and Table 2. The microcapsules and other concrete materials were mixed in a concrete mixer. The finished cube specimen is shown in Figure 1.

## 3. Test Method and Evaluation Indices

### 3.1. Test Method

A three-factor, three-level experimental design was adopted; the orthogonal experimental design is shown in Table 3. For the convenience of comparison, a group of blank concrete specimens was used in addition to the nine groups of specimens shown in the table. The design strength of the concrete was C30. The prepared specimens were cured under standard conditions, and the curing times were 7 d and 28 d. The ultimate compressive strength of the concrete specimens was measured at 7 d and 28 d, and the loading rate was 5–8 kN/s. The other concrete samples were pre-damaged, and the damage load was 90%, 80%, and 70% of the ultimate compressive strength. The cube specimens with a damage degree of 100% and 90% after drying were tested by ultrasonic flaw detection. Subsequently, the four types of concrete cubes with different damage degrees were soaked in water and left to heal for 30 d. A universal testing machine was used to measure the compressive strength of concrete, and an NM-4A non-metallic ultrasonic testing analyzer (Kangkerui Company) was used for the ultrasonic flaw detection.

### 3.2. Evaluation Indices

Due to the lack of a unified standard for the evaluation index of self-healing concrete, the most commonly used method is to compare the performance indices of concrete before and after damage. Some references used acoustic emission as the method to monitor and inspect mechanical behavior of cementitious structures [26,27,28,29,30,31]. In this paper, the recovery rate of the compressive strength and the recovery rate of the sound speed of the concrete were used. The expressions are as follows:

(1) $$R(\sigma )=\frac{\sigma_{healed}}{\sigma_{initial}}\;\times \;100\%\;ƞ\;(u)\;=\;\frac{u_{healed}\;-\;u_{control}}{u_{control}}\;\times \;100\%$$

where *R*(σ) is the strength recovery rate of the healed specimen, $\sigma_{healed}$ is the strength of the healed specimen, and $\sigma_{initial}$ is the initial strength of the specimen. *ƞ* (u) is the sound velocity recovery rate after healing, *u*_*healed*_ is the wave velocity of the healed concrete cube, *u*_*control*_ is the sound velocity before concrete damage.

## 4. Influencing Factors on Compressive Strength

The ultimate compressive strength of the orthogonal test and ordinary concrete specimens is listed in Table 4.

Table 5 and Table 6 list the analysis of variance results of the ultimate compressive strength of the cube specimens. The results in Table 5 and Table 6 show that the compressive strength of the test specimens is optimal at 5% microcapsule content, 50% sodium silicate concentration, and 10% sodium fluosilicate content, and the combination level is A3B2C1. The *F-*value of the compressive strength shows that the order of influence of the factors in the test is C > B > A. The most important factor affecting the compressive strength of the specimens is the percentage of sodium fluosilicate, followed by the proportion of microcapsules and the microcapsule content. The three factors can have a significant impact on the compressive strength of the concrete. The confidence level of the lowest influencing factor (microcapsule dosage) on the compressive strength of concrete is 60%. The *F*-value of the proportion of sodium fluorosilicate is significantly higher than that of the other two factors, and the confidence level exceeds 95%.

The order of influence of the factors on the compressive strength at 28 d is B > C > A, and the influence of factor B is similar to that of factor C. Compared with the concrete aged for 7 d, factor A has a lower *F*-value and B has a higher *F*-value for the concrete cured for 28 d, indicating that the strength of the microcapsules may have a larger influence on the concrete aged for 28 d. In addition, the significant decrease in the *F*-value of factor C is due to the retarding effect of sodium fluosilicate. An increase in its dosage results in a more pronounced retarding effect for the concrete cured for 7 d and a decrease in the retarding effect for the concrete cured for 28 d.

**Figure 2:**
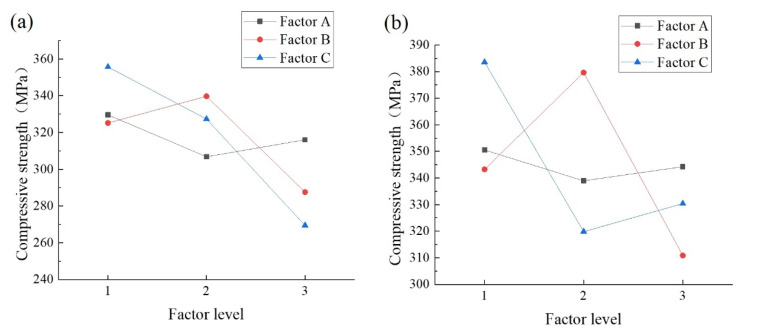
*K*-value of the ultimate compressive strength of the cube specimens. (**a**) Compressive strength at 7 d; (**b**) Compressive strength at 28 d.

A comparison of the *K*-values in Figure 2 indicates that for factor A, the strength is the highest at a microcapsule content of 1%, indicating that an increase in the microcapsule content reduces the concrete strength, but the strength reduction is not significant at contents of 3% and 5%. For factor B, the concrete strength is the highest at 50% sodium silicate. For factor C, the compressive strength of concrete decreases with the sodium fluosilicate content, which is consistent with the conclusion that a high content of sodium fluosilicate reduces the compressive strength of concrete.

## 5. Influencing Factors on the Recovery Rate of the Compressive Strength

### 5.1. Variance Analysis of Influencing Factors on Strength Recovery

The recovery rate of the compressive strength according to the ultimate compressive strength of the cube specimens before and after healing is listed in Table 7 and Table 8. The strength recovery rates of the nine experimental groups are higher than that of the control group, demonstrating the effectiveness of the microcapsule self-healing concrete system.

**Figure 3:**
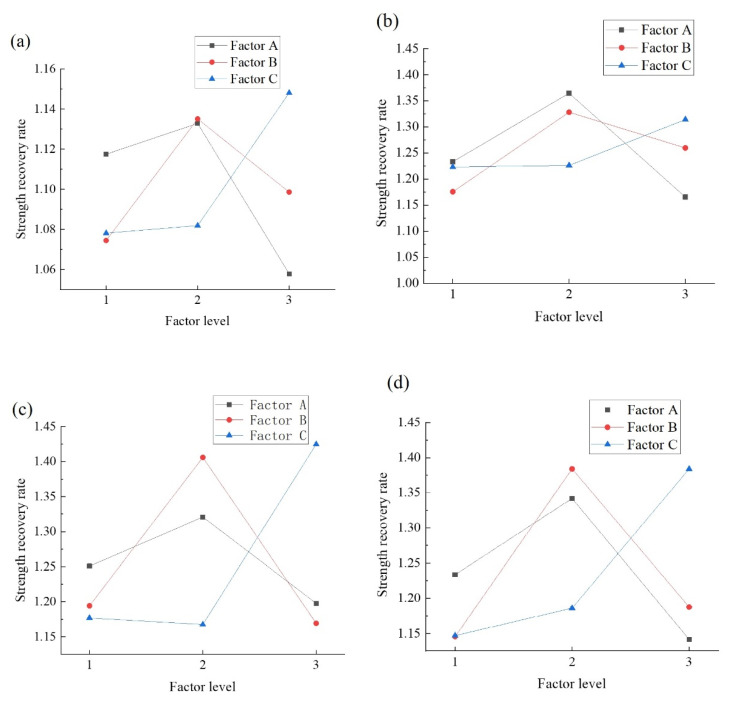

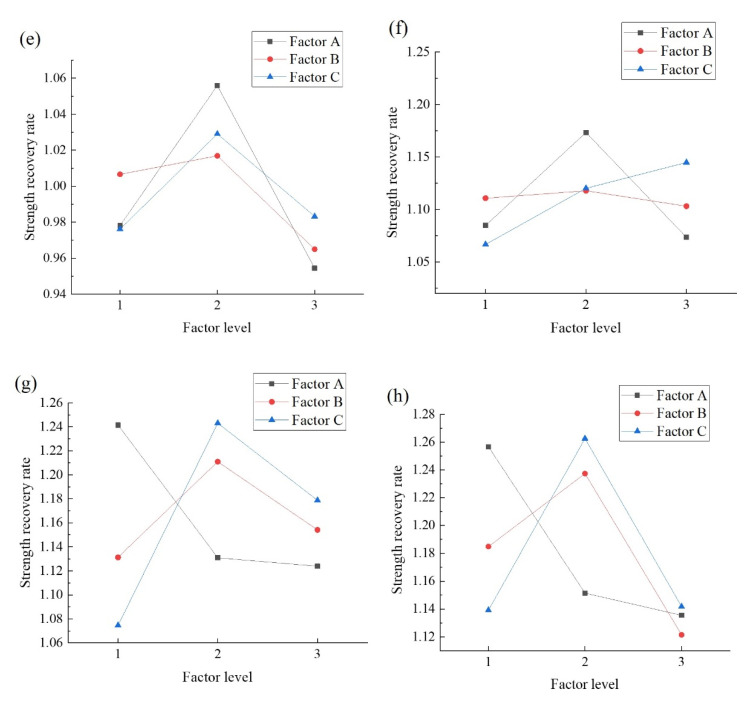
*K*-value of the compressive strength recovery rate for various factors. (**a**) 100% damage of specimens at 7 d. (**b**) 90% damage of specimens at 7 d. (**c**) 80% damage of specimens at 7 d. (**d**) 70% damage of specimens at 7 d. (**e**) 100% damage of specimens at 28 d. (**f**) 90% damage of specimens at 28 d. (**g**) 80% damage of specimens at 28 d. (**h**) 70% damage of specimens at 28 d.

The *K*-values (Figure 3) indicate that the maximum recovery rate is obtained after 7 d of curing, and the maximum healing rate of the specimens with 100% and 90% damage loads at 28 d is obtained at 3% dosage. In the 80% and 70% damage conditions, the recovery rate decreases with an increase in the dosage. The healing ability of the microcapsules is poor due to the insufficient microcapsule content at 1% dosage. However, at 5% dosage, the damage to the concrete is greater, indicating that the self-healing performance is impaired in pre-damaged specimens. The self-healing performance is lower at a microcapsule content of 5% than 3% at 80% and 70% damage loads at 28 d of curing. The reason may be that there are fewer cracks under a low damage load, especially for concrete aged for 28 d. Therefore, there are fewer microcapsules with crack damage. As a result, the healing rate is reduced. Besides, factor A has a negligible influence on the compressive strength, whereas the 100% and 90% damage loads are the primary factors influencing the healing rate for the two curing conditions. Therefore, the optimal factor level of factor A in the system is 3%.

The *K*-value indicates that a 50% sodium silicate content is the optimum factor level for the microcapsule self-healing concrete. Although Portland cement provides strength recovery, it is insufficient, leading to a decrease in the self-healing performance. The strength of the microcapsules with a 70% sodium silicate content is relatively low; thus, there are more cracks. In this case, sodium silicate acts as a binder but cannot fill the cracks. Therefore, the strength recovery performance of the self-healing microcapsules consisting entirely of sodium silicate was relatively low. In addition, the concentration of the core had a substantial influence on the compressive strength and recovery rate at 7 d but a negligible effect at 28 d (the *F*-value was about 1) and was low at a concentration of 70%. Therefore, the optimum factor level of the sodium silicate content is 50%.

The influence of factor C (sodium fluosilicate content) on the recovery rate depends on the curing time. At 7 d of curing, the recovery rate increases with an increase in the sodium fluosilicate content due to its retarding, resulting in a substantial reduction in the concrete strength aged for 7 d and an increase in the recovery rate in the later period. At aging for 28 d, the optimum factor level of C2 is 15%. The reason is the same as for factor B, i.e., the healing effect at a low dosage cannot compensate for the cracks, but the damage is similar at a high dosage.

### 5.2. Influence of Damage Loads on the Significance of Various Factors

The *F*-values of the compressive strength recovery rate after healing at aging for 7 d and 28 d are listed in Table 9 and Table 10. Unlike at pre-damage loads of 70% and 80%, at 100%, the most influential factor is the microcapsule content at 7 d and 28 d. The reasons are as follows. Under complete or almost complete failure, there are more cracks in the concrete and the microcapsules, and the self-healing efficiency largely depends on the number of damaged microcapsules. Therefore, the microcapsule content determines the number of broken microcapsules, i.e., the self-healing rate. Therefore, under a high damage load, a high *F-*value is obtained and vice versa. This finding demonstrates the effectiveness of the self-healing performance of the microcapsule self-healing concrete system.

### 5.3. The Optimum Factor Combination to Optimize the Recovery Rate

The variance analysis shows that in most cases, the *F*-values of the factors are greater than 1, and the confidence level is greater than 50%, indicating that all three factors affect the self-healing efficiency of concrete. The influence of the sodium fluorosilicate content is higher under a lower damage load because there are fewer broken microcapsules, and the sodium silicate may dissolve in water. The more sodium fluorosilicate is added, the higher the reaction rate is, and the greater the strength recovery is.

The variance analysis and *K*-values of the compressive strength and compressive strength recovery rate indicate that the optimum factor combination in the microcapsule self-healing concrete system is A2B2C2, (a microcapsule content of 3%, a sodium silicate content of 30%, and a sodium fluorosilicate content of 15%). This combination provides the optimum self-healing effect of the concrete without reducing the concrete strength under different damage loads.

## 6. Influencing Factors on the Acoustic Performance Recovery Rate under High Load Damage Conditions

### 6.1. Influencing Factors on Acoustic Performance Recovery Rate

As the effect of the sound velocity of concrete is negligible at damage loads of 80% and 70%, the ultrasonic wave velocity was only tested for concrete specimens with 100% and 90% pre-damage. The test results are listed in Table 11 and Table 12.

**Figure 4:**
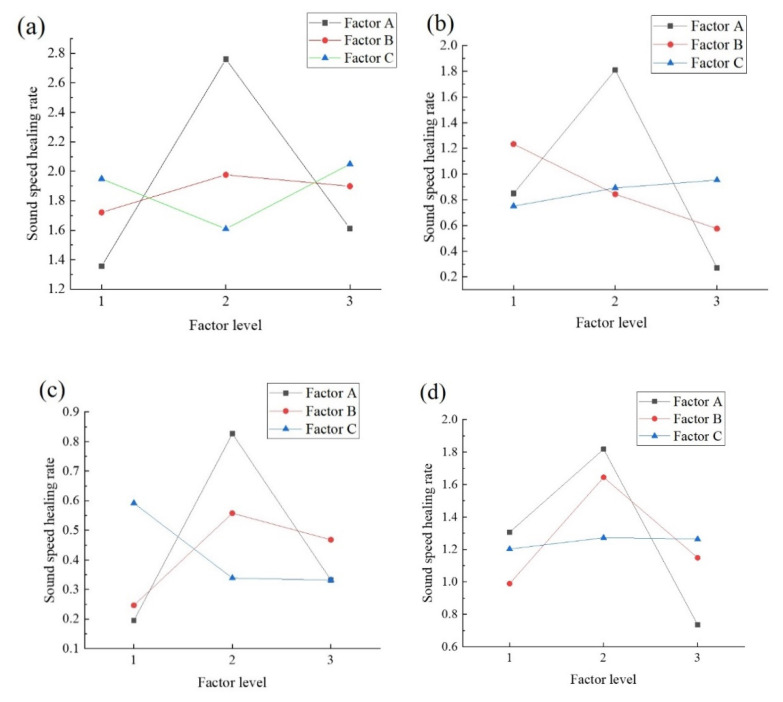
*K*-value of the sound speed recovery rate for various factors. (**a**) 100% damage and aging for 7 d. (**b**) 90% damage and aging for 7 d. (**c**) 100% damage and aging for 28 d. (**d**) 90% damage and aging for 28 d.

For the sound speed recovery rate, the largest *F*-values are obtained for the microcapsule content, which is consistent with the concrete healing rate under a high damage load. The confidence level for the microcapsule dosage is more than 75%, whereas that of the other two factors is almost zero. Figure 4 shows that the *K*-values of factors B and C are relatively gentle, and the maximum sound speed recovery rate is obtained at 3% dosage; thus, the optimal factor level of the sound speed recovery rate of factor A is 3%.

### 6.2. Comparison of Acoustic Performance Recovery and Compressive Strength Recovery

The most important factor affecting the sound speed recovery rate is the degree of crack filling or shrinkage of the concrete after cracks have formed, which leads to the shrinkage of internal cracks and holes, restoring the propagation speed of the ultrasonic wave. As this study focuses on concrete with extensive damage, the most important factor affecting the sound speed recovery rate is the amount of the healing material, i.e., the microcapsule content. Sodium silicate is an adhesive that bonds with concrete and affects the extension and expansion of concrete cracks. The concrete also shrinks in the natural environment; thus, a change in the sodium silicate content will have an effect, especially under low damage loads. Sodium fluorosilicate is a curing agent of concrete. Although sodium fluorosilicate and sodium silicate have a curing effect, the main focus of this study is the concrete strength. The bonding effect of sodium fluorosilicate is the same at high and low dosages, but different curing effects have different influences on the concrete strength. Therefore, the influence of the sodium fluosilicate content on the sound speed recovery rate is negligible.

There are some differences between the recovery rate of the compressive strength of the specimens and the sound speed recovery rate. For example, the other two factors have only a negligible influence. Only the sodium silicate content under a 90% damage load has a low confidence level. The characterization of acoustic properties has distinct advantages and disadvantages compared with the compressive strength recovery rate. An ultrasonic velocity test can be carried out without destroying the specimen, and it is highly sensitive to cracks, and the strength recovery is be affected by the concrete strength. There are also disadvantages. Due to the strong randomness of natural fractures in concrete [32], crack extension and expansion are difficult to predict, and the damage degree of each part is often difficult to distinguish using only the pre-damage load. The results may differ for the same pre-damage load of concrete at the same location due to the different extension of cracks. Therefore, the sound velocity change obtained during flaw detection may have disadvantages.

## 7. Analysis of Healing Morphology

### 7.1. Healing Morphology

**Figure 5:**
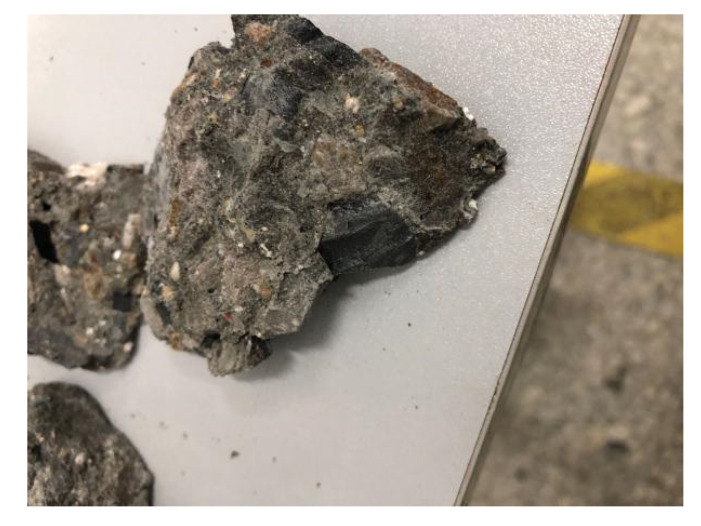
Damaged concrete fragments.

Some fragments of the completely damaged specimen are shown in Figure 5. The microcapsules break at the concrete section, and the broken microcapsules are randomly distributed in the mortar matrix. In the ideal state, if the core material has sufficient self-healing ability in the micro-crack stage, a micro-crack healing effect occurs when the healing material diffuses into the microcracks.

**Figure 6:**
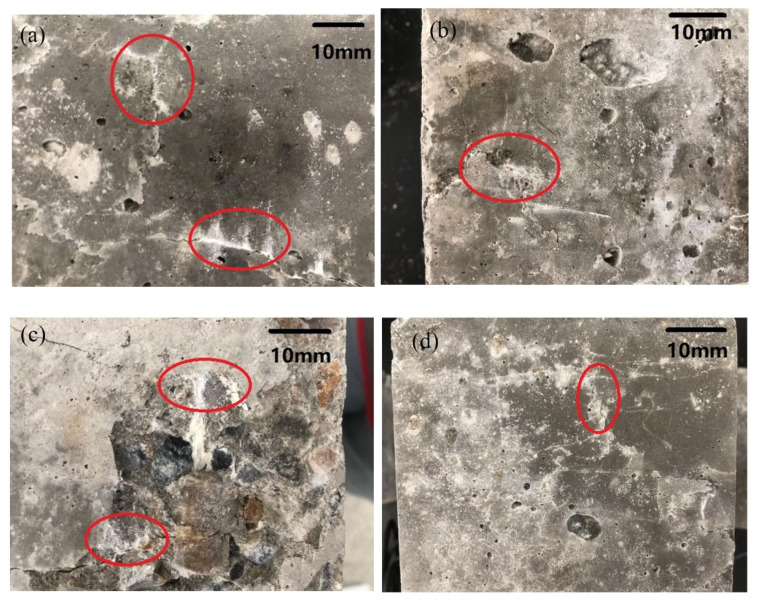
Healed specimen. (**a**)After healing in A2B2C3 group. (**b**) After healing in A1B2C2 group. (**c**) After healing in A3B3C2 group. (**d**) After healing in A2B2C2 group.

The images of the concrete specimens with 100% damage after immersion healing are shown in Figure 6. A large number of traces of the released core material are observed (red positions). A small number of macro-cracks are completely filled by powdery crystals, and most of them appear at the crack location.

### 7.2. Micromorphology and EDS Analysis of the Broken Microcapsules in the Concrete

**Figure 7:**
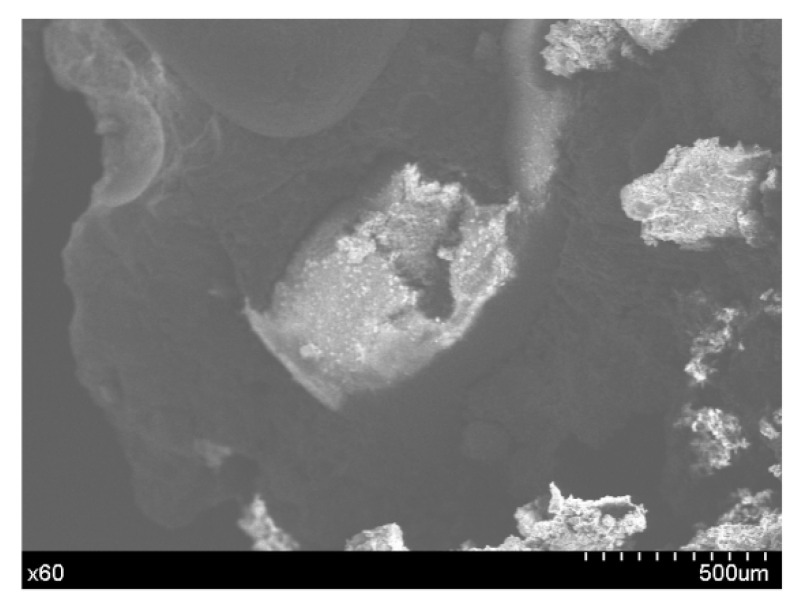
Morphology of the damaged microcapsules in the concrete.

**Figure 8:**
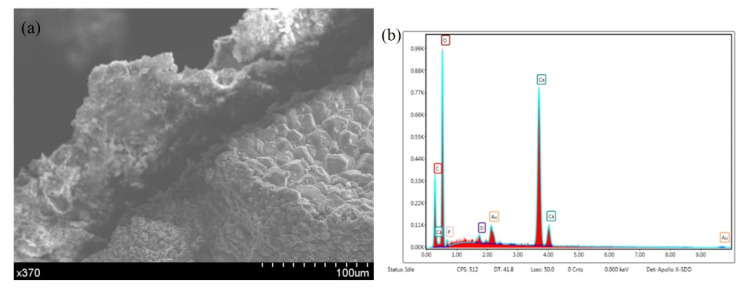
Microcapsule self-healing wall material in the concrete after crushing. (**a**) Micromorphology. (**b**) EDS results.

The micromorphology of the damaged microcapsules in the concrete is shown in Figure 7. The combined effect of the microcapsules and the concrete is good. The microcapsules are broken and deformed due to damage to the concrete. EDS analysis of the capsule wall material (Figure 8) indicates large amounts of carbon in the wall material, except for the residual calcium carbonate. The analysis shows that the carbon in the wall material is ethyl cellulose. The wall material of the microcapsules is wrapped around the core, and the surface is in close contact with the concrete. The analysis shows that the microcapsule self-healing concrete system provides an excellent self-healing performance.

### 7.3. Microstructure and EDS Analysis of the Specimens

**Figure 9:**
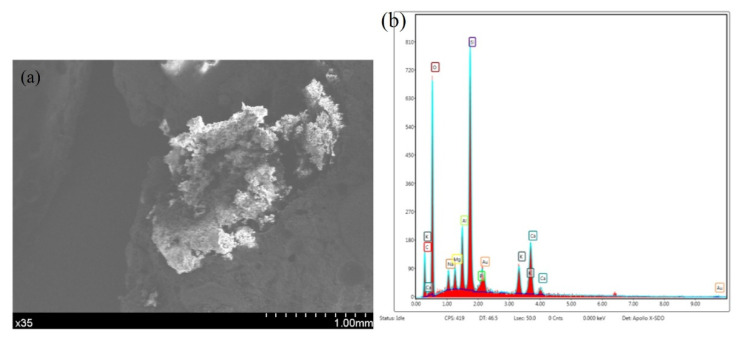
The micromorphology and EDS composition of the sample after healing. (**a**) Micromorphology. (**b**) EDS results.

**Figure 10:**
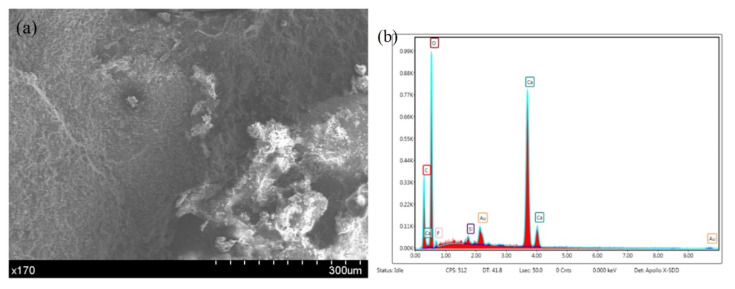
Microstructure and EDS results of the sample after healing. (**a**) Micromorphology. (**b**) EDS results.

The EDS analysis of the specimens (Figure 9) and the results in Figure 10 indicate that the sodium ion content is zero, the primary component of the microcapsules is silicon, and there are small amounts of F and K. Studies of concrete containing sodium silicate as the healing material have shown that sodium silicate and sodium fluosilicate generate sodium fluoride and silica gel and react with carbon dioxide as follows [33]:

(2) $$\mathrm{Si}\;+\;\mathrm{NaF}——{\mathrm{SiO}}_{2}\mathrm{NaF}\cdot H_{2}O\;+\;H_{2}O\;\mathrm{and}\;{\mathrm{Na}}_{2}O\cdot n{\mathrm{SiO}}_{2}\;+\;2nH_{2}O\;+\;{\mathrm{CO}}_{2}——{\mathrm{Na}}_{2}{\mathrm{CO}}_{3}\;+\;\mathrm{nSi}{\left(\mathrm{OH}\right)}_{4}$$

**Figure 11:**
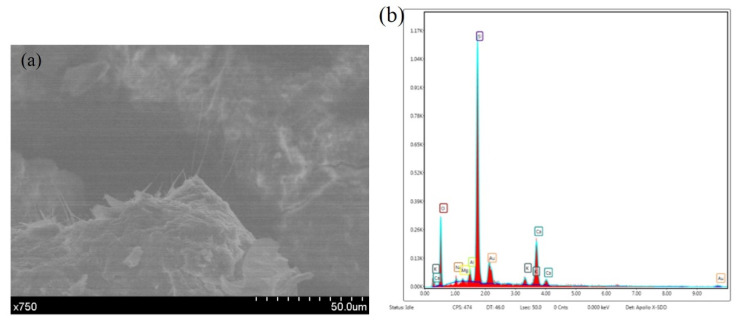
The micromorphology and EDS results of the cracks. (**a**) Micromorphology. (**b**) EDS results.

Sodium fluoride is soluble and sodium carbonate is easily soluble in water, resulting in sodium loss. In addition, Figure 8 shows that as well as large amounts of calcium carbonate, there are small amounts of F and Si, which may be a small amount of SiO_2_NaF. Finally, as shown in Figure 11, a 750-fold increase in the number of fractures occurred in the capillary substance. The EDS analysis of the substance reveals large amounts of silicon and oxygen. The reason is that Si (OH) _4_ gel is required for the reaction.

## 8. Conclusions

In this paper, the strength recovery and acoustic performance of microcapsule self-healing concrete were used as evaluation indices to assess the damage to the microcapsule self-healing system. An orthogonal experimental design with three factors was used. The optimum factor combination of the system and the best mix proportion of the microcapsule self-healing concrete were determined. The following conclusions were drawn.

(1) The evaluation indices showed that the self-healing performance was better for the microencapsulated self-healing concrete than the ordinary concrete. The healing effect of the self-healing concrete decreased with an increase in the pre-damage load, and the sound speed recovery rate increased with an increase in the damage degree.
(2) All three factors affected the healing rate. In contrast, the proportion of sodium silicate and the sodium fluosilicate dosage had a negligible influence on the sound speed recovery rate at a high damage degree.
(3) The microcapsule content was the highest under a high damage load and vice versa. The influence of the sodium silicate content on the compressive strength and compressive strength recovery rate of the self-healing concrete increased, followed by a decrease. The curing agent had a more significant effect on the concrete at 7 d than at 28 d of curing.
(4) The optimum combination of factors of the microcapsule self-healing system was 3% microcapsules, 30% sodium silicate, and 15% sodium fluosilicate.

## Figures and Tables

**Table 1:** Physical properties of the cement.

Specific Surface Area (m^2^/g)	Initial Setting Time (min)	Final Setting Time (min)	Water Requirement of Normal Consistency (%)	Boiling Stability	3 d Compressive Strength (MPa)	28 d Compressive Strength (MPa)
345	140	260	26.5	qualified	27.1	42.5

**Table 2:** Chemical components of the cement.

Components	SiO_2_	Al_2_O_3_	Fe_2_O_3_	CaO	MgO	SO_3_	K_2_O	Na_2_O	LOI
Content	21.6%	4.3%	2.6%	65.8%	1.2%	1.6%	0.7%	0.4%	1.8%

**Table 3:** Orthogonal test design.

No.	Dosage of Microcapsule (A)	Weight Ratio of Sodium Silicate (B)	Dosage of Curing Agent (C)	Factor Combination
1	1%	30%	10%	A1B1C1
2		50%	15%	A1B2C2
3		70%	20%	A1B3C3
4	3%	30%	15%	A2B1C1
5		50%	20%	A2B2C3
6		70%	10%	A2B3C1
7	5%	30%	20%	A3B1C3
8		50%	10%	A3B2C1
9		70%	15%	A3B3C2

**Table 4:** Ultimate compressive strength of concrete.

No.	7 d Compressive Strength (Unit: kN)	28 d Compressive Strength (Unit: kN)
No microcapsule	277.75	326.53
1	366.5	404.23
2	307.58	342.2
3	264.63	305.13
4	301.27	315.38
5	272.1	375.87
6	311.03	325.57
7	271.67	310.13
8	389.35	420.8
9	287.07	301.87

**Table 5:** The *F*-values of the ultimate compressive strength of the cube specimens at 7 d.

Source of Variation	Sum of Squares	Freedom	Mean Square Error	*f*-Value
A	788.21962	2	394.10981	1.055454
B	2006.6314	2	1003.3157	2.686952
C	11,522.109	2	5761.0546	15.42852
error e	705.39216	2	352.69608	
error ∆e	1493.6118	4	373.40294	
Sum	16,515.964			

**Table 6:** The *F*-values of the ultimate compressive strength of the cube specimens at 28 d.

Source of Variation	Sum of Squares	Freedom	Mean Square Error	*f*-Value
A	201.57396	2	100.78698	0.198066
B	7101.2284	2	3550.6142	6.977649
C	6996.9584	2	3498.4792	6.875193
error e	1833.8476	2	916.92381	
error ∆e	2035.4216	4	508.85539	
Sum	18,169.032			

**Table 7:** Recovery rate of compressive strength after 7 d of curing.

Pre-Damage Load	100%	90%	80%	70%
0	101.96%	102.33%	109.96%	112.63%
1	108.85%	118.32%	114.39%	115.28%
2	109.88%	124.52%	125.26%	127.38%
3	116.54%	127.12%	135.59%	127.39%
4	109.04%	103.07%	119.37%	114.22%
5	123.44%	145.84%	167.34%	173.70%
6	107.39%	100.51%	109.53%	114.67%
7	104.46%	111.27%	124.51%	114.11%
8	107.22%	118.08%	129.18%	114.14%
9	105.67%	120.25%	105.65%	114.21%

**Table 8:** Recovery rate of compressive strength after 28 d of curing.

Pre-Damage Load	100%	90%	80%	70%
0	94.96%	99.37%	101.58%	103.73%
1	98.15%	105.72%	104.54%	113.36%
2	101.05%	108.74%	142.30%	144.97%
3	94.24%	110.97%	125.59%	118.65%
4	110.76%	113.13%	118.69%	126.54%
5	107.65%	113.10%	111.87%	108.36%
6	98.36%	100.77%	108.72%	110.53%
7	93.08%	109.36%	116.16%	115.53%
8	96.36%	103.51%	109.11%	117.86%
9	96.90%	109.19%	111.91%	107.25%

**Table 9:** *F*-values of the strength recovery rate after healing at aging for 7 d.

	Damage Load	100%	90%	80%	70%
Source of Variation					
A		1.856179	3.797547	1.183875	1.098678
B		1.099399	2.158215	5.259925	1.770761
C		1.827456	0.988696	6.616161	1.761606

**Table 10:** *F*-values of the strength recovery rate after healing at aging for 28 d.

	Damage Load	100%	90%	80%	70%
Source of Variation					
A		5.138002	1.866152	1.449658	0.972559
B		1.375637	0.067652	0.561392	0.755348
C		1.501199	0.835639	2.410909	1.11525

**Table 11:** Ultrasonic sound speed recovery rate (age of 7 d).

	Degree of Damage	100%		90%	
Source of Variation					
Index		Sum of Squares of *K*	*F*	Sum of squares of *K*	*F*
A		0.045717	2.572828	0.022859	4.231512
B		0.001542	0.086644	0.000813	0.773694
C		0.004881	0.274612	0.002440	0.079657

**Table 12:** Ultrasonic sound speed recovery rate (age of 28 d).

	Degree of Damage	100%		90%	
Source of Variation					
Index		Sum of Squares of *K*	*F*	Sum of squares of *K*	*F*
A		0.038651	1.932322	0.040472	10.471671
B		0.011320	0.565969	0.014791	3.826955
C		0.008062	0.403081	0.000195	0.050535

## Data Availability

No new data were created or analyzed in this study. Data sharing is not applicable to this article.
